# Consecutive episodes of heart and kidney failure in an “otherwise” healthy young man

**DOI:** 10.1186/s12882-019-1414-y

**Published:** 2019-06-20

**Authors:** V. Esposito, D. Catucci, M. Colucci, M. Torreggiani, F. Grosjean, C. Esposito

**Affiliations:** 1 Unit of Nephrology, ICS S. Maugeri SpA SB, Pavia, Italy; 2 Unit of Nephrology, IRCCS Policlinico San Matteo, Via Camillo Golgi 19, 27100 Pavia, Italy; 3 University of Pavia, Via S. Maugeri 10, 27100 Pavia, Italy

**Keywords:** Sarcoidosis, Granuloma, Heart failure, Acute kidney injury, Kidney biopsy, Steroid treatment

## Abstract

**Background:**

Acute renal failure is a rare occurrence in a patient with an unremarkable past medical history and should always lead to an in depth clinical study. The occurrence in the same healthy young subject, of consecutive episodes of heart failure and of acute renal failure is an even rarer event and should prompt diagnostic tests and restrict the diagnostic hypotheses.

**Case presentation:**

We present the case of a 28 year-old man who, while waiting to undergo assessment for a mild chronic kidney disease, was diagnosed with decompensated dilated cardiomyopathy and placed on diuretics and β-blockers. After few weeks he developed a non oligoanuric acute renal failure with a slight elevation of serum calcium. Renal biopsy proved suggestive for renal sarcoidosis; thus the hypothesis of systemic sarcoidosis with cardiac and renal involvement was possible avoiding further delay in initiation of therapy.

**Conclusions:**

Cardiac sarcoidosis is usually silent but the majority of cases are diagnosed when cardiac symptoms are present in a patient with systemic sarcoidosis. Renal involvement with granulomatous interstitial nephritis is also quite rare and can be an unexpected finding at kidney biopsy. This case highlights the need to evaluate thoroughly clinical problems that do not fit in a specific scenario and emphasizes the importance of performing a kidney biopsy in case of kidney failure of unknown etiology.

## Background

Sarcoidosis is a multisystem inflammatory disease of unknown origin characterized by the presence of non caseating epithelioid granulomas in the involved organs [1]. Usually the disease affects young and middle aged adults with a benign course and spontaneous remission in up to 2/3 of cases. However, in 1/3 of cases the disease may lead to organ impairment [1]. Although lungs, lymph nodes and skin are the most frequently involved organs, sarcoidosis may affect every organ [2]. The incidence of cardiac and renal sarcoidosis is not known although necropsy series report the presence of granulomas in the heart and kidney in up to 48% of patients with sarcoidosis [3, 4]. Though a well-known disease, sarcoidosis is difficult to recognize because symptoms, even if very severe, are quite unspecific especially for cardiac and renal localization that may mimic other more common diseases.

## Case report

A 28 year-old male was found to have a slightly increased serum creatinine (1.7 mg/dl) during the admission to ER because of abdominal pain. Since both his past medical history and an abdominal ultrasound performed on admission were unremarkable he was discharged with the indication to see a Nephrologist. At the Nephrology outpatient clinic, because of the reduced eGFR and of the presence of proteinuria (300 mg/24 h), an hospitalization for further investigations was planned.

While waiting for the admission, a couple of weeks later, the patient was admitted to the emergency room in another hospital for abdominal pain and dyspnea. On admittance the ECG tracing performed showed sinus rhythm with diffuse repolarization abnormalities. Further exams revealed an increase of troponin (54 ng/ml) and transaminases levels (GOT 50 U/L; GPT 125 U/L) and a cardiac ultrasound showed a severe left ventricular dysfunction with right heart failure. Patient was thus admitted to the coronary intensive care unit. During his hospital stay the possibility of acute myocarditis was excluded because of the absence of a recent viral syndrome and the negativity of IgM antibodies against the viruses most commonly affecting the cardiovascular system and because of the low inflammatory indexes. An abdominal ultrasound demonstrated abdominal and pleural effusions, regular size and morphology of spleen and liver, no pancreatic changes; both kidneys had normal size but showed irregular margins and a reduced thickness of the cortex, no hydronephrosis nor stones were observed. A magnetic resonance demonstrated an increased volume of the cardiac chambers with a severe reduction of the function of both ventricles (EF20%), however no signs of reduced myocardial perfusion, valvular defects or other tissue changes such as inflammation, fibrosis or edema could be demonstrated. A chest CT scan confirmed the presence of pleural effusion and showed parenchymal atelectasias mainly involving the lower lobes of the lungs. The patient was discharged after 11 days on diuretics (furosemide 100 mg/daily), B-blocker (carvedilol 37.5 mg/daily), acetyl salicylic acid (Aspirin 100 mg/day) and an Ace inhibitor (Ramipril 5 mg/day) with a diagnosis of dilated cardiomyopathy and severe left ventricular dysfunction complicated with cardiogenic shock and anasarca. Few days later an EKG ergometric test revealed no areas of inducible ischemia. A further admission to the Cardiology Unit to define the effect of treatment was performed a week later. Cardiac ultrasound confirmed the left ventricular dysfunction (EF 23%), a coronary angiography was negative and a right chamber catheterism and endomyocardic biopsy demonstrated no specific lesion nor inflammatory infiltrate. The patient was then admitted to our Nephrology Unit, as planned, to evaluate the cause of the chronic kidney disease. On admission the patient appeared healthy and well oriented, his blood pressure was 110/60 mmHg, heart rate 56 bpm, physical examination was unremarkable except for a 1/6 intensity systolic heart murmur better audible on mitral valve area. ECG tracing showed sinus bradycardia. Laboratory studies revealed an increased serum creatinine (1.87 mg/dl), eGFR 48 ml/min/1.73 m^2^, hypercalcemia (serum calcium 11.5 mg/dl), hemoglobin 15.1 g/dl, sodium 135 mEq/L, potassium 4.37 mEq/L, phosphate 3.1 mg/dl, serum albumin 4.07 g/dl. The patient denied use of vitamin D analogs thus in consideration of the unexplained hypercalcemia other exams were performed. Thyroid hormones, calcitonin, vitamin D, serum ACE, phosphate and calcium urinary excretion rate were all within the normal range. PTH was lower than 3 pg/ml. To rule out multiple myeloma serum protein electrophoresis, serum and urine immunofixation, and spine and skull x-ray were performed and did not show any pathological changes.

**Fig. 1:**
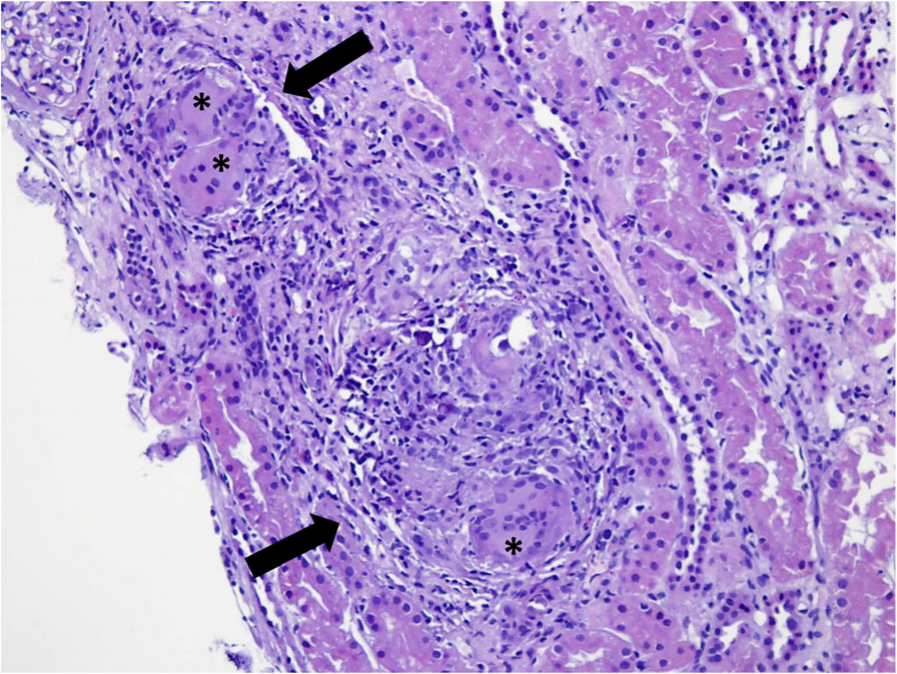
Photomicrograph of a section of the patient’s kidney biopsy showing two non caseating granulomas (arrows) containing giant multinucleated cells (asterixes) surrounded by inflammatory infiltrate. A normal glomerulus is shown in the left upper corner. H&E staining, magnification: × 200

**Fig. 2:**
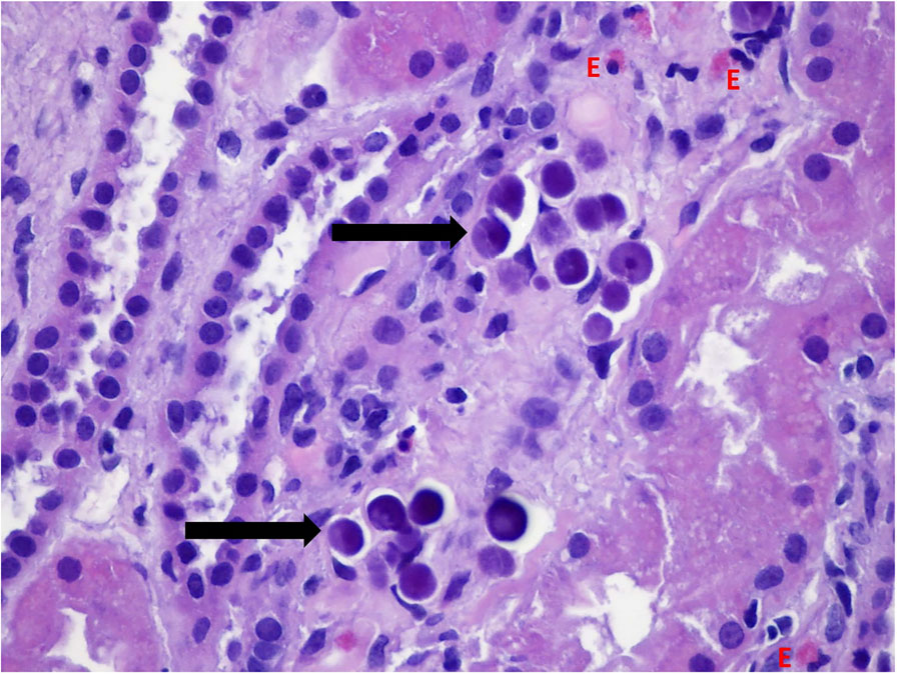
Photomicrograph of a section of the patient’s kidney biopsy showing calcium crystals in the tubules (arrows) and infiltrating eosinophyls (E). H&E staining, magnification × 400

Renal biopsy was finally performed without complications and the patient was discharged after 24 h. While waiting for the renal biopsy pathology report, medications were not modified, but for an increase of the dose of furosemide because of the hypercalcemia. The renal biopsy contained eight glomeruli none of which was globally or partially sclerotic and no pathological changes were observed at glomerular level, vessels were also unremarkable. Tubulointerstitium was the only affected compartment with a moderate to severe inflammatory infiltrate, some calcium crystals in the tubular lumen and two granulomas with multinucleated giant cells (Figs. 1 and 2). No necrosis could be observed within the granulomas. A diagnosis of granulomatous interstitial nephritis due to sarcoidosis was made.

When the patient was called to discuss his kidney biopsy, his serum creatinine had further increased (3.5 mg /dl), eGFR 22 ml/min/1.73 m^2^ and so had his serum calcium (13.3 mg/dl), serum albumin 4.06 g/dl. For this reason, he was hospitalized again and treated with pulse methylprednisolone (250 mg/day for three days) and then with maintenance dose prednisone (50 mg/day). Serum creatinine decreased in a few days and so did his serum calcium. Since the episode of acute kidney injury the patient has been followed at our unit and at the unit of Cardiology. Renal function returned to normal (serum creatinine 1.1 mg/dl, eGFR 91 ml/min/1.73 m^2^) and heart function markedly improved over the following two years (EF 49% vs 37% vs 32% vs 23%). The diuretic and cardiologic therapy was significantly reduced to only 25 mg of furosemide and a small dose of beta blocker. The steroid was gradually tapered to 5 mg/day of prednisone for one year and then stopped.

## Discussion and conclusions

Sarcoidosis is an inflammatory disease of unknown etiology characterized by non-caseating granulomas and by variable clinical course which can affect every organ and system [1]. Although the diagnosis of sarcoidosis can be made on clinical grounds when several specific clinical findings are present, in the majority of cases it is not so easy, especially when the affected organs are the ones less commonly involved, such as heart and kidney [5]. Cardiac and renal sarcoidosis are rarely diagnosed. Of note the involvement of heart and kidney is not so rare in the course of the disease since according to autopsy studies its prevalence is at least 20 to 30% [4] nonetheless, less than 5% of the patients suffer from clinical cardiac or kidney sarcoidosis [6, 7].

Cardiac sarcoidosis may be completely silent or induce several cardiac changes which may vary from conduction abnormalities to sudden death [8]. Despite the highly variable presentation, the prognosis of cardiac sarcoidosis is severe; thus it is imperative to make a diagnosis and start a treatment as soon as possible. The diagnosis of cardiac involvement in sarcoidosis is extremely challenging [9] because of a lack of a gold standard test. Even the myocardial biopsy, which was also performed in our patient, may not be diagnostic due to patchy distribution of the disease [10]. Algorithm and diagnostic criteria have been developed and considered as established criteria till recently but they have proved to be insensitive for detecting cardiac involvement [11]. These studies however have shown that CMR and PET have a central role in the diagnosis and the management of cardiac sarcoidosis. In the present study CMR and endomyocardial biopsy were performed to determine the cause of the dilated cardiomyopathy but did not yield any results. A heart biopsy may result negative in case of sarcoidosis because sarcoid granulomas are less commonly located in the right ventricle where biopsies are commonly taken. 18F-FDG PET, which enables visualization of sarcoid lesions in various organs including the heart, was not performed in our patient and a diagnosis of dilated cardiomyopathy was made even though the majority of the conditions causing a cardiomyopathy could be ruled out based on medical and familial history and demographic data. No attempt was made to administer steroid as an ex adiuvantibus treatment probably because the possibility of a case of cardiac sarcoidosis never occurred to the cardiologists.

When the patient was admitted to the Nephrology Unit the presence of unexplained hypercalcemia [12], the reduction of eGFR and the recent episode of HF led to further investigations and to perform the kidney biopsy even though there were no strong indications but only a mild increase of serum creatinine and of proteinuria [13, 14]. All the known causes of hypercalemia such as primary hyperparathyroidism, vitamin D intoxication, malignancies, thiazides, familial hypocalciuric hypercalcemia, milk alkali syndrome, were ruled out [12]. Physical examination, serum angiotensin converting enzyme (sACE) levels and chest x ray, considered central in the work-up for the diagnosis of sarcoidosis were negative or normal. The presence of non caseating granulomas with epithelioid-type multinucleated giant cells and associated interstitial nephritis and calcification at the kidney biopsy allowed the diagnosis in our patient [15]. The rapid improvement of renal function and the progressive amelioration of cardiac ejection fraction with steroid treatment confirmed our diagnosis of cardiac and renal sarcoidosis ruling out other granulomatosis diseases [16]. To date this is the first report of consecutive severe cardiac and acute renal injury due to sarcoidosis, a disease that should always be considered in young patients with unexplained multiple organ diseases. Cardiac and renal involvement is rare but it may lead to severe organ impairement if not promptly recognized. Biopsy may not be diagnostic because of the sampling issues, as occurred in the cardiac involvement of our patient. However a trial of steroid therapy may be justified in patients with strong clinical suspicion of cardiac or renal sarcoidosis even with a non diagnostic biopsy [17].

## Data Availability

All the information about the case report are available from the corresponding author on reasonable request.

## Abbreviations

18F-FDG PET: 18F fluorodeossiglucose Positron Emission Tomography
ACE: Angiotensin Converting Enzyme
ALT: Alanine Aminotransferase
AST: Aspartate Aminotransferase
CMR: Cardiac Magnetic Resonance
CT: Comtputed Tomograph
ECG: Electrocardiogram
EF: Ejection Fraction
eGFR: estimated Glomerular Filtration Rate
ER: Emergency Room
HF: Heart Failure
PET: Positron emission Tomography
PTH: Parathyroid Hormone
